# Supplementary material for: Causal Relationship between Aging and Anorexia Nervosa: A White-Matter-Microstructure-Mediated Mendelian Randomization Analysis
Source: Biomedicines. 2024 Aug 16;12(8):1874. doi: 10.3390/biomedicines12081874 (PMC11351342; doi:10.3390/biomedicines12081874)
Supplement: Supplementary file 1 [file biomedicines-12-01874-s001.zip › Supplementary Figure/Figure S4/Singe SNP plot of MR analysis for TL to ubm-b-2031.pdf]

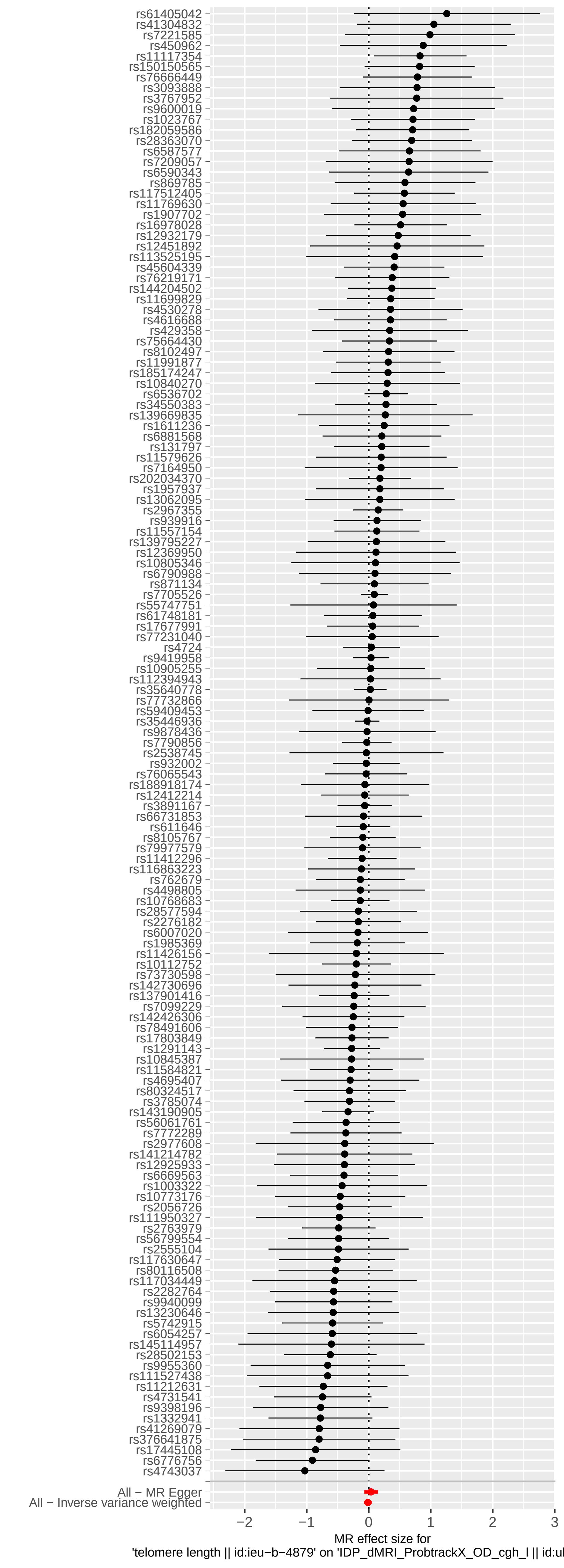

rs61405042
rs41304832
rs7221585
rs450962
rs11117354
rs150150565
rs76666449
rs3093888
rs3767952
rs9600019
rs1023767
rs182059586
rs28363070
rs6587577
rs7209057
rs6590343
rs869785
rs117512405
rs11769630
rs1907702
rs16978028
rs12932179
rs12451892
rs113525195
rs45604339
rs76219171
rs144204502
rs11699829
rs4530278
rs4616688
rs429358
rs75664430
rs8102497
rs11991877
rs185174247
rs10840270
rs6536702
rs34550383
rs139669835
rs1611236
rs6881568
rs131797
rs11579626
rs7164950
rs202034370
rs1957937
rs13062095
rs2967355
rs939916
rs11557154
rs139795227
rs12369950
rs10805346
rs6790988
rs871134
rs7705526
rs55747751
rs61748181
rs17677991
rs77231040
rs4724
rs9419958
rs10905255
rs112394943
rs35640778
rs77732866
rs59409453
rs35446936
rs9878436
rs7790856
rs2538745
rs932002
rs76065543
rs188918174
rs12412214
rs3891167
rs66731853
rs611646
rs8105767
rs79977579
rs11412296
rs116863223
rs762679
rs4498805
rs10768683
rs28577594
rs2276182
rs6007020
rs1985369
rs11426156
rs10112752
rs73730598
rs142730696
rs137901416
rs7099229
rs142426306
rs78491606
rs17803849
rs1291143
rs10845387
rs11584821
rs4695407
rs80324517
rs3785074
rs143190905
rs56061761
rs7772289
rs2977608
rs141214782
rs12925933
rs6669563
rs1003322
rs10773176
rs2056726
rs111950327
rs2763979
rs56799554
rs2555104
rs117630647
rs80116508
rs117034449
rs2282764
rs9940099
rs13230646
rs5742915
rs6054257
rs145114957
rs28502153
rs9955360
rs111527438
rs11212631
rs4731541
rs9398196
rs1332941
rs41269079
rs376641875
rs17445108
rs6776756
rs4743037
All − MR Egger
All − Inverse variance weighted
−2
−1
0
1
2
3
MR effect size for
'telomere length || id:ieu−b−4879' on 'IDP_dMRI_ProbtrackX_OD_cgh_l || id:ul
